# Management of Busulfan-Induced Lung Injury in Pediatric Patients with High-Risk Neuroblastoma

**DOI:** 10.3390/jcm13195995

**Published:** 2024-10-08

**Authors:** Sveva Castelli, Anne Thorwarth, Claudia van Schewick, Anke Wendt, Kathy Astrahantseff, Annabell Szymansky, Marco Lodrini, Simon Veldhoen, Alexander Gratopp, Marcus A. Mall, Angelika Eggert, Hedwig E. Deubzer

**Affiliations:** 1 Department of Pediatric Oncology and Hematology, Charité—Universitätsmedizin Berlin, Corporate Member of Freie Universität Berlin and Humboldt-Universität zu Berlin, Campus Virchow Klinikum, Augustenburger Platz 1, 13353 Berlin, Germany; sveva.castelli@charite.de (S.C.); annabell.szymansky@charite.de (A.S.); marco.lodrini@charite.de (M.L.);; 2 Department of Pediatric Respiratory Medicine, Immunology and Critical Care Medicine, Charité–Universitätsmedizin Berlin, Campus Virchow Klinikum, Augustenburger Platz 1, 13353 Berlin, Germany; 3 German Cancer Consortium (DKTK), Partner Site Berlin and German Cancer Research Center (DKFZ), Im Neuenheimer Feld 280, 69120 Heidelberg, Germany; 4 Department of Pediatric Radiology, Charité–Universitätsmedizin Berlin, Campus Virchow Klinikum, Augustenburger Platz 1, 13353 Berlin, Germany; 5 German Center for Lung Research (DLZ), Associated Partner Site Berlin, 89337 Berlin, Germany; 6 German Center for Child and Adolescent Health (DZKJ), Partner Site Berlin, 89337 Berlin, Germany; 7 Berlin Institute of Health (BIH), Anna-Louisa-Karsch-Straße 2, 10178 Berlin, Germany; 8 Experimental and Clinical Research Center (ECRC) of Charité and Max-Delbrück-Center of Molecular Medicine in the Helmholtz Association, Lindenberger Weg 80, 13125 Berlin, Germany; 9 Max-Delbrück Center of Molecular Medicine in the Helmholtz Association, Robert-Rössle-Straße 10, 13125 Berlin, Germany

**Keywords:** busulfan, busulfan-induced lung injury, high-dose chemotherapy, neuroblastoma, pediatric cancer, pharmacogenomics, restrictive lung disease, therapeutic drug monitoring

## Abstract

**Background/Objectives**: Integrating the cytotoxic drug busulfan into a high-dose chemotherapy regimen prior to autologous hematopoietic stem cell rescue in patients with high-risk neuroblastoma has improved the survival of children battling this deadly disease. Busulfan-induced toxicities can, however, be severe. Here, we describe the diagnosis and successful treatment of acute pulmonary injury by total-body-weight-adjusted busulfan therapy in two children with high-risk neuroblastoma. **Case series**: Patient 1 developed life-threatening biphasic acute respiratory failure on days +60 and +100 after busulfan therapy, requiring intubation and invasive mechanical ventilation. Despite intensive anti-inflammatory and immunomodulatory therapy, including systemic corticosteroids, topical inhalation regimens, azithromycin, nintedanib and extracorporal photopheresis, patient 1 required extended intensive care measures and non-invasive respiratory support for a total of 20 months. High-resolution computed tomography showed diffuse intra-alveolar and interstitial patterns. Patient 2 developed partial respiratory failure with insufficient oxygen saturation and dyspnea on day +52 after busulfan therapy. Symptoms were resolved after 6 months of systemic corticosteroids, topical inhalation regimens and azithromycin. High-resolution computed tomography showed atypical pneumonic changes with ground-glass opacities. While both patients fully recovered without evidence of pulmonary fibrosis, cancer therapy had to be paused and then modified until full recovery from busulfan-induced lung injury. **Conclusions**: Busulfan-induced lung injury requires prompt diagnosis and intervention. Symptoms and signs are nonspecific and difficult to differentiate from other causes. Therapeutic busulfan drug level monitoring and the identification of patients at risk for drug overdosing through promoter polymorphisms in the glutathione S-transferase alpha 1 gene encoding the main enzyme in busulfan metabolism are expected to reduce the risk of busulfan-induced toxicities.

## 1. Introduction

Busulfan is a key cytotoxic drug used in high-dose chemotherapy followed by autologous hematopoietic stem cell transplantation in patients with high-risk neuroblastoma [1,2,3]. The busulfan/melphalan high-dose chemotherapy (BuMel HD) regimen has become the standard of care in the SIOPEN high-risk (HR) strategy following the demonstration of its superiority over the carboplatin, etoposide and melphalan high-dose chemotherapy (CEM HD) regimen in the HR-NBL1/SIOPEN randomized phase 3 clinical trial (NCT01704716) [4]. In the HR-NBL1/SIOPEN trial, 50% of patients receiving the BuMel HD regimen, and only 38% receiving the CEM HD regimen, survived for 3 years without event. At the 5-year mark, 45% of patients receiving the BuMel HD regimen versus 33% receiving the CEM HD regimen survived without an event, while 5-year overall survival was 54% for the BuMel HD patient subgroup and 41% for the CEM HD patient subgroup [4].

Busulfan can, however, cause major toxicities, including sinusoidal obstruction syndrome and restrictive lung disease [2,5,6]. Busulfan-induced lung injury is a rare but critical complication that can trigger life-threatening acute or chronic respiratory failure [7,8]. The literature on managing busulfan-induced lung injury in the pediatric patient population is scarce, with only few reports existing [7,8,9]. Here, we share the clinical, radiological and laboratory features as well as the pulmonological and oncological management of two patients with high-risk neuroblastoma and busulfan-induced lung injury.

## 2. Case Report for Patient 1

**Figure 1:**
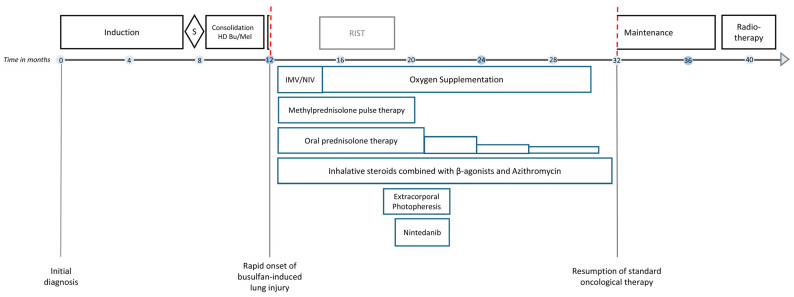
Schematic diagram illustrating the medical interventions for busulfan-induced lung injury in patient 1 and its consequences for cancer therapy. Induction polychemotherapy was administered in accordance with the rapid platinum-containing induction schedule (carboplatin, cisplatin, vincristine, etoposide, cyclophosphamide) according to the SIOPEN HR-NBL1 protocol (NCT01704716). Consolidation therapy consisted of weight-adjusted high-dose chemotherapy with i.v. busulfan and melphalan (HD BuMel). Maintenance immunotherapy included five cycles of the monoclonal anti-GD2 antibody dinutuximab beta. Radiotherapy of the preoperative tumor bed was administered at 21.6 Gy. Acute respiratory failure and acute respiratory distress syndrome due to busulfan-induced lung injury required invasive mechanical ventilation (IMV) including nitric oxide supplementation followed by prolonged weaning employing non-invasive ventilation (NIV). A total of eight methylprednisolone pulses were administered. Oral prednisolone (2 mg/kg/d) was given in between pulses and was carefully tapered. RIST, molecularly targeted multimodal therapy consisting of metronomic courses of rapamycin/dasatinib and irinotecan/temozolomide. S, surgery. The red dashed line indicates the interruption of the standard oncological treatment of patient 1 due to busulfan-induced lung injury.

A 5-year-old boy with metastasized high-risk neuroblastoma (primary thoracic tumor, multiple osteomedullar metastases; genetics: diploid *MYCN*, wildtype *ALK*) was transferred to our center with very good partial response at the end of induction and completely resected primary tumor for consolidation therapy (Figure 1) according to recommendations of the pan-European SIOPEN Group [10] and German Society of Pediatric Oncology and Hematology [11]. Busulfan dosages were adjusted to the total body weight, followed by autologous hematopoietic stem cell transplantation (10.48 × 106 CD34 + cells/kg body weight). Neutrophil and thrombocyte engraftment was achieved on days +12 and +19, respectively, and the patient was discharged in good clinical condition on day +30. Restaging on day +45 demonstrated stable disease according to the revised International Neuroblastoma Response Criteria [12]. At this timepoint, there was no evidence of lung injury during physical examination and monitoring of the vital parameters. Maintenance therapy was started with the GD2-directed dinutuximab beta monoclonal antibody on day +60.

**Figure 2:**
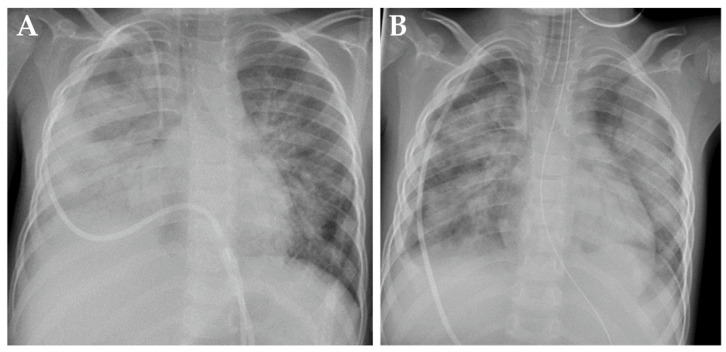
Chest X-rays from patient 1 demonstrating signs of busulfan-induced lung injury on day +62 (**A**) and day +74 (**B**) after high-dose chemotherapy.

On day 62+, patient 1 developed rapid respiratory deterioration, requiring intubation and invasive mechanical ventilation (IMV) with 100% oxygen and 20 ppm nitric oxide, the latter due to refractory hypoxemia. A chest X-ray showed pathological signs compatible with right-sided pleuropneumonia and overall pulmonary edema (Figure 2A). Immunotherapy was halted. No pathogens were identified in bronchoalveolar lavage, respiratory swabs, blood, urine or stool samples. Broad empirical antibiotic and antifungal therapy was administered immediately after the collection of biospecimens for infectious disease work-up. Assuming a pulmonary cytokine-release syndrome, anti-interleukin 6 therapy with tocilizumab was administered without clear positive effect. After 5 days of IMV and intravenous hydrocortisone (100 mg/m^2^/d), patient 1 was extubated and continued with non-invasive high-flow nasal cannula therapy.

**Figure 3:**
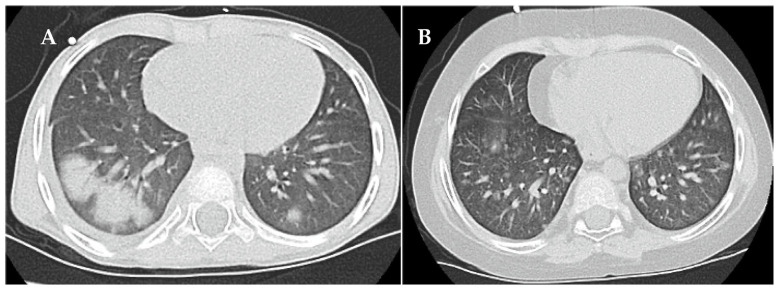
High-resolution computed tomography findings of busulfan-induced lung injury in patient 1 at diagnosis (**A**) and at follow-up eight months after start of the anti-inflammatory and immunomodulatory therapy (**B**).

On day +74, respiratory failure with rapidly evolving tachydyspnoea recurred. The chest X-ray now demonstrated bilateral pulmonary infiltrates and pleural effusions (Figure 2B). Patient 1 was re-intubated and placed on IMV as an emergency measure for 6 days. Systemic inflammatory parameters were again high but without a causal pathogen (extensive testing). Empiric antibiotic and antifungal regimens were nevertheless applied. High-resolution computed tomography (HRCT) showed increased ground-glass opacification and consolidation, particularly in both lower lobes (Figure 3A). Busulfan-induced lung injury was the leading differential diagnosis at this stage, and a methylprednisolone pulse was administered (Figure 1). This anti-inflammatory therapy improved the respiratory situation, eventually enabling successful extubation. Patient 1 required non-invasive ventilation followed by high-flow nasal cannula therapy and continuous oxygen supplementation via a nasal cannula for 20 months (Figure 1).

Treatment of busulfan-induced lung injury included a total of eight methylprednisolone pulses and continuous prednisolone therapy at a dose of 2 mg/kg/d, which could only be tapered from month 4 onwards (Figure 1). The systemic corticosteroid therapy, causing the full spectrum of Cushing’s disease, was combined with inhalative corticosteroids, inhalative short-acting β-agonists and oral azithromycin therapy in line with the graft-versus-host lung stage 1 treatment protocol used in the allogeneic setting, as we considered the evidence for successful anti-inflammatory therapy of the lung high when following these recommendations. In total, 15 cycles of extracorporal photopheresis were also performed to employ its immunomodulatory effects [13,14,15] against the as yet not fully understood molecular cascades causing lung injury (Figure 1). HRCT performed at 8 months after the clinical onset of busulfan-induced lung injury showed that the consolidation had resolved (Figure 3B). Spirometry presented a restrictive pattern with stepwise amelioration over time. To delay the potential initiation and progression of fibrotic processes in the lung, oral therapy with the small-molecule tyrosine kinase inhibitor, nintedanib, was administered for 3 months [16]. The patient fully recovered with no remaining signs of busulfan-induced lung injury in spirometry. Standard oncological therapy (radiotherapy of the preoperative thoracic tumor bed followed by immunotherapy) could not be administered. Therefore, four cycles of RIST therapy (NCT01467986) were administered as a bridging therapy, which needed to be discontinued to treat infectious complications, most likely due to the extended immunosuppression caused by the combination with corticosteroid therapy. Patient 1 resumed standard oncological therapy after recovery from the busulfan-induced lung injury and tolerated the dinutuximab beta-based maintenance therapy very well.

## 3. Case Report for Patient 2

**Figure 4:**
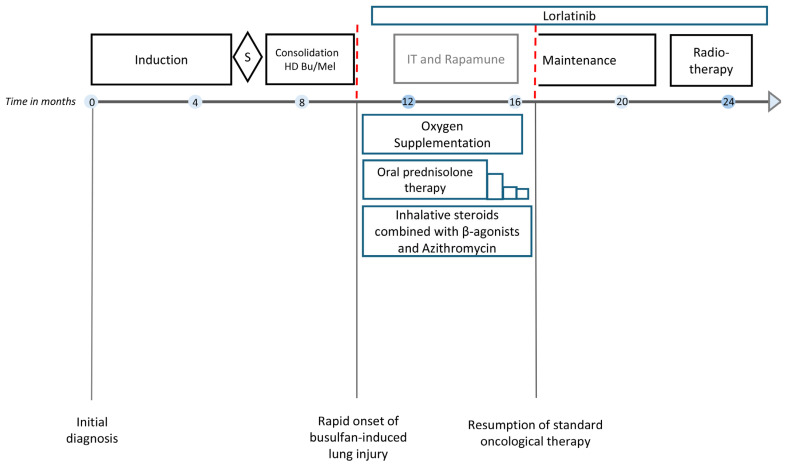
Schematic diagram illustrating the medical interventions for busulfan-induced lung injury in patient 2 and its consequences for the treatment of high-risk neuroblastoma according to the GPOH NB2017 Guidance. Induction polychemotherapy was administered in accordance with the GPOH induction schedule (3xN5 cycle: vindesine, cisplatin, etoposide; 3xN6 cycle: vincristine, dacarbazine, ifosfamide, doxorubicin). Consolidation therapy consisted of weight-adjusted high-dose chemotherapy with i.v. busulfan and melphalan (HD Bu/Mel). Maintenance immunotherapy included five cycles with the monoclonal anti-GD2 antibody dinutuximab beta. Radiotherapy was given at 21.6 Gy to the preoperative tumor bed. Acute partial respiratory failure due to busulfan-induced lung injury required continuous oxygen supplementation. Oral prednisolone was started at 2 mg/kg/d, then gradually tapered. IT and rapamune, multimodal therapy consisting of metronomic courses of rapamune and irinotecan/temozolomide modified from the RIST scheme (NCT01467986), with the multityrosine kinase inhibitor dasatinib replaced by third-generation ALK tyrosine kinase inhibitor lorlatinib; S, surgery. The red dashed line indicates the interruption of the standard oncological treatment of patient 2 due to busulfan-induced lung injury.

A 2.5-year-old boy was diagnosed with metastasized high-risk neuroblastoma in our center (primary tumor: bilateral abdominal sympathetic chains with infiltration of left kidney; metastases: osteomedullar lesions, lymph nodes; genetics: diploid *MYCN*, *ALK* R.1275Q mutation with allele frequency in tumor: 21.4%, blood plasma: 40.1%, bone marrow plasma: 40.5%). Patient 2 received therapy according to GPOH NB2017 Guidance for the high-risk group [11] including the BuMel HD regimen with total-body-weight-adjusted dosing of intravenous busulfan as also recommended by the pan-European SIOPEN Group [10] (Figure 4). Patient 2 had achieved a complete remission at the end of induction and developed hepatic sinusoidal obstruction syndrome on day +6 of the consolidation therapy, which was successfully treated with defibrotide [17,18]. Neutrophil engraftment was achieved on day +13 and platelet engraftment, slightly delayed, on day +60.

**Figure 5:**
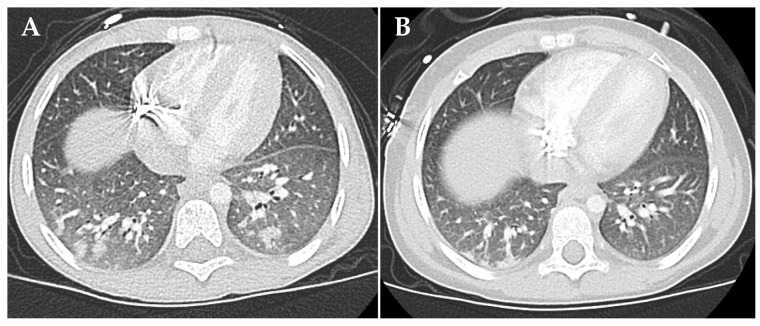
High-resolution computed tomography findings of busulfan-induced lung injury in patient 2 at diagnosis (**A**) and six months after start of the anti-inflammatory therapy (**B**). (**A**) Shown are atypical pneumonic changes, characterized by ground-glass opacification and patchy consolidation in the lower lobes. (**B**) Shown is the regression of consolidations and opacities. There is no evidence of pulmonary fibrosis.

At day +52 after autologous stem cell transplantation, patient 2 developed partial respiratory failure with dyspnea and need for continuous low-level oxygen. The chest X-ray showed moderate bilateral infiltrates and pulmonary venous congestion. HRCT imaging revealed atypical pneumonic changes, characterized by ground-glass opacification and patchy consolidation in the lower lobes (Figure 5A). An empirical antibiotic and antifungal therapy was initiated. No pathogen was identified in a comprehensive infectious diagnostic work-up. At disease onset, radiological signs and echocardiographic findings did not suggest pulmonary veno-occlusive disease. Systemic prednisolone therapy (2 mg/kg/d) stabilized respiratory status. Topical treatment comprising inhalative corticosteroids and short-acting β-agonists in combination with azithromycin was initiated. Patient 2 required continuous oxygen supplementation for 6 months (Figure 4). A modified version of the four-compound RIST therapy (NCT01467986, experimental arm; dasatinib multityrosine kinase inhibitor substituted by the third-generation ALK tyrosine kinase inhibitor, lorlatinib) was employed as a bridging oncological therapy for 6 months. The therapy was paused according to standard oncological procedures in case of (respiratory tract) infections. HRCT findings 6 months after partial respiratory failure onset showed regression of consolidations and opacities and no evidence of pulmonary fibrosis (Figure 5B). Patient 2 resumed and completed the standard oncological treatment regimen and has remained in first complete remission until the date of publication (Figure 4).

## 4. Discussion

Diagnosing drug-induced lung disease relies on the temporal association between exposure to the causative agent and onset of respiratory signs and symptoms [19]. Excluding other potential causes of lung damage is the most critical factor for accurate diagnosis [20,21]. Busulfan-induced lung injury is a diagnosis of exclusion due to the lack of unique clinical features, specific biomarkers and histological findings [22,23]. Thorough conduct of comprehensive diagnostic testing is essential, particularly to rule out infections frequently caused by atypical pathogens in immunocompromised patients [7]. Busulfan-induced lung injury is a complex and potentially life-threatening condition, exemplified in the clinical courses of our two patients, that requires prompt diagnosis and treatment [7,8,19]. Cancer therapy must, unfortunately, be interrupted in most cases, thus substantially increasing the risk for progressive disease or relapse in the patients affected.

Pulmonary toxicity associated with busulfan can range from mild interstitial pneumonitis to end-stage pulmonary fibrosis and occurs at variable times following drug administration [7,19]. Clinicians must be vigilant for respiratory symptoms in patients who receive busulfan and employ a multidisciplinary approach in case of complications. Regular monitoring of pulmonary function and imaging facilitate detection of very early signs of drug-induced lung injury [24]. HRCT is the preferred imaging modality to diagnose and monitor pulmonary toxicity over time [24,25]. Patterns associated with busulfan toxicity include ground-glass opacities, peripheral and peribronchial consolidation, centrilobular nodules and reticulation [23]. Persisting HRCT changes suggest drug-induced lung damage [23,25]. Pulmonary function tests (spirometry, lung diffusion testing) are required to measure pulmonary damage severity and assess therapy response [7,19,26].

Cell-mediated immune reactions, including cytokine release, are suspected to be at the root of busulfan-induced lung injury, although the pathophysiology has not yet been fully elucidated. Systemic anti-inflammatory corticosteroid therapy is, therefore, essential [7,27], with the severity of respiratory pathophysiology and response determining cumulative dose and therapy duration [26]. Apart from corticosteroids and supportive measures, no other evidence-based therapies for drug-induced pneumopathies exist to date.

The extended therapeutic approach applied in this case series is in line with treatment protocols for other lung diseases with substantial inflammatory activity, including pulmonary graft-versus-host disease, bronchiolitis obliterans syndrome and interstitial lung diseases. Topical therapy with inhalative corticosteroids was combined with short-acting β-agonists in line with the expert opinion for treating chronic inflammation [28,29]. The well-characterized anti-inflammatory and immunomodulatory properties of azithromycin [30] have been shown to reduce the incidence of infectious exacerbations in patients with chronic obstructive pulmonary disease [31] and non-cystic fibrosis bronchiectasis [32].

Extracorporeal photopheresis, which exposes mononuclear cells to ultraviolet A irradiation in the presence of a photosensitizing agent, acts by inducing apoptosis and dendritic cell differentiation from monocytes as well as modifying cytokine profiles and T-cell subpopulations to promote immune tolerance [13,14,15]. As excessive inflammatory processes are hypothesized to trigger busulfan-induced lung injury, with the eventual onset of tissue fibrosis [33], we also added multiple cycles of extracorporeal photopheresis to the treatment schedule for patient 1. We hypothesized that its immunomodulatory effects could attenuate the decline in pulmonary function [33,34,35] and help reduce the corticosteroid dose necessary to suppress inflammatory cascades in the lung [36]. The small-molecule tyrosine kinase inhibitor, nintedanib, has previously been shown to inhibit fibrotic remodeling of lung tissue in preclinical models [37,38]. Nintedanib effectively decelerated progression of fibrosis and respiratory function decline in adults with interstitial lung disease [16], and an acceptable safety profile was demonstrated in the phase 3 InPedILD trial (NCT04093024) in children with fibrosing interstitial lung disease [39]. To prevent fibrotic lung disease, we also treated patient 1 with nintedanib [16].

The pharmacokinetic properties of busulfan have been intensely studied in recent years [6,40,41]. Inter-patient variability of busulfan plasma concentration is high, risking stronger toxicity in patients with high plasma concentrations [42]. Busulfan clearance is particularly variable in the pediatric population and is higher in young children [6,43]. Several studies have highlighted the benefits of therapeutic drug monitoring approaches to busulfan dosing [41,44,45,46], which has become the standard of care in the allogeneic transplant setting. While total-body-weight-based dosing of intravenous busulfan has achieved the desired therapeutic drug level in the past [15,47], implementing advanced therapeutic drug monitoring may improve the toxicity profile of the BuMel HD regimen, which improves survival of patients with high-risk neuroblastoma [4]. The hepatic busulfan metabolism is mediated by glutathione S-transferases [48,49,50]. Functional promoter polymorphisms in the main enzyme, glutathione S-transferase alpha 1 (GSTA1), have been shown to influence enzyme activity and, thus, pharmacokinetics and toxicity [51,52,53,54]. Pharmacogenomic data obtained prior to implementation of the BuMel HD regimen may add information to better understand busulfan exposure and the double-edged sword of busulfan efficacy or toxicity in individual patients when associated with pharmacokinetics.

## 5. Conclusions

The busulfan drug used in the high-dose chemotherapy regimen against high-risk neuroblastoma can cause major toxicities including restrictive lung disease. This case series describes the multimodal therapy of busulfan-induced lung toxicity with varying severity, and its control and complete resolution of symptoms, in pediatric patients with high-risk neuroblastoma. Early diagnosis as well as systemic and topical anti-inflammatory and immunomodulatory therapies present the cornerstone of its management. Therapeutic drug monitoring combined with pharmacogenetics may contribute to reduce busulfan toxicity in this vulnerable patient group.

## Data Availability

No new data were created or analyzed in this study. Data sharing is not applicable to this article.
